# A case of laryngeal cancer induced by exposure to asbestos in a construction site supervisor

**DOI:** 10.1186/s40557-016-0114-3

**Published:** 2016-08-08

**Authors:** Sooyong Roh, Soyong Park, Gyeong Tae, Jaechul Song

**Affiliations:** 1 Department of Occupational and Environmental Medicine, Hanyang University, Seoul, Republic of Korea; 2 Department of Occupational and Environmental Medicine, Kangbuk Samsung Medical Center, Seoul, Republic of Korea; 3 Department of Otolaryngology Head and Neck Surgery, Hanyang University, Seoul, Republic of Korea

**Keywords:** Laryngeal neoplasms, Occupational exposure, Construction industry, Asbestos

## Abstract

**Background:**

Construction site supervisors are exposed to many chemicals, dusts, and metals including asbestos. Asbestos is a hazardous chemical that is carcinogenic. Laryngeal cancer is not a rare disease in Korea. The most common causes of this disease are tobacco and alcohol, and representative occupational cause is asbestos. However, up to now, no case of laryngeal cancer induced by asbestos has been reported in Korea. In this study, we report such a case in a construction site supervisor.

**Case presentation:**

A 60-year-old man who had been experiencing hoarseness for 2 months was diagnosed with laryngeal cancer. The pathologic diagnosis was squamous cell carcinoma in situ, based on examination of a biopsy specimen obtained by resection of the lesion. The patient had been exposed to asbestos for 38 years at construction sites where he worked until diagnosed with laryngeal cancer. He had been exposed to asbestos when demolishing buildings and inspecting materials.

**Conclusion:**

The patient in this case worked with construction materials including asbestos and supervised construction for 38 years, and was thus exposed to asbestos at construction sites. Much of the asbestos was highly concentrated especially during demolition processes. We therefore consider the laryngeal cancer of this patient to be a work-related disease.

## Background

Laryngeal cancer account for 2 ~ 5 % of all cancers and 25 ~ 30 % of head and neck cancers [1]. About 85 ~ 95 % of laryngeal cancers are squamous cell carcinomas; 34 % are located in the supraglottis, 51 % in the glottis, 1 % in the subglottis, and 14 % occur in more than one location [2].

Laryngeal cancer has the same risk factors as cancers of the head and neck. More than 90 % of laryngeal cancers occur in male over 40 years old [3]. The reason for this is that men are more exposed to the most important risk factors, namely smoking and drinking [4]; however the difference between the male and female incidence rates has decreased in the last 60 years as female smoking has increased. There are no differences in laryngeal cancer incidence rates due to ethnicity. The risk of the disease is proportional to the severity of smoking and drinking and to the length of exposure. The risk slowly decreases after quitting smoking but takes nearly 20 years to become the same as in nonsmokers [5, 6]. Also smoking and drinking have synergistic effects [7]. Although this depends on the location, glottic cancer tends to be related to smoking and supraglottic cancer is to drinking [2]. Other than this, chronic stimulation of the larynx for example by pharyngolaryngeal reflux and human papillomavirus (HPV) are also risk factors [8–10]. Occupational causes of laryngeal cancer include asbestos which is a representative carcinogen, long term exposure to inorganic dust at workplaces and working in rubber manufacturing [11]. Recently the International Agency for Research on Cancer (IARC) reported that there is reliable evidence that laryngeal cancer and ovarian cancer can be caused by asbestos [12]. However there are no reports of such cases in Korea.

We encountered a case of laryngeal cancer in a construction site supervisor exposed to asbestos. We describe the case below, along with a literature review to help assess whether the patient’s laryngeal cancer was caused by occupational asbestos exposure and to contribute to future case diagnoses and judgments occupational disease.

## Methods

The patient’s occupational history, work content, environment, social history, past history, and present illness were recorded in detail through history taking and his occupational insurance medical care expenses document, work-relatedness evaluation, medical records, etc. were examined after receiving the consent and cooperation of patient and guardian. Next domestic and foreign publications were reviewed focusing on the epidemiology, pathophysiology and risk factors of laryngeal cancer, with emphasis on the relevance to this disease of occupational exposure. After this two occupational and environmental medicine physicians visited the workplace of the patient with approval. The visit was carried out to establish the patient’s work pattern, type of work and working environment.

## Case presentation

Patient: M/60

Chief complaint: hoarseness, sensation of foreign body in the throat for 2 months

**Fig. 1:**
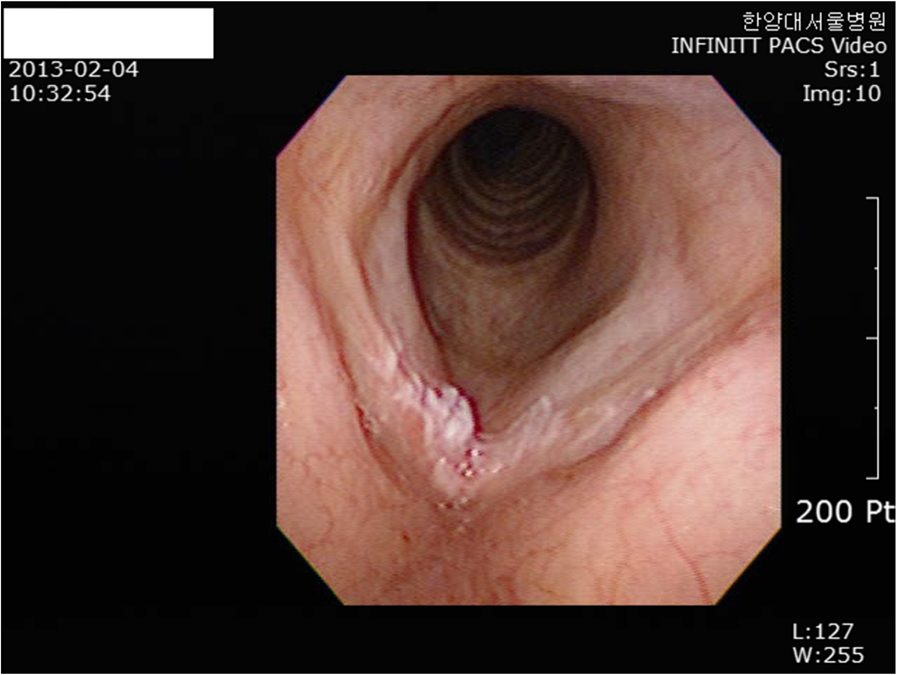
Laryngoscopy (02/02/2013). A lesion, an irregular surface, in the employee’s right vocal cord

**Fig. 2:**
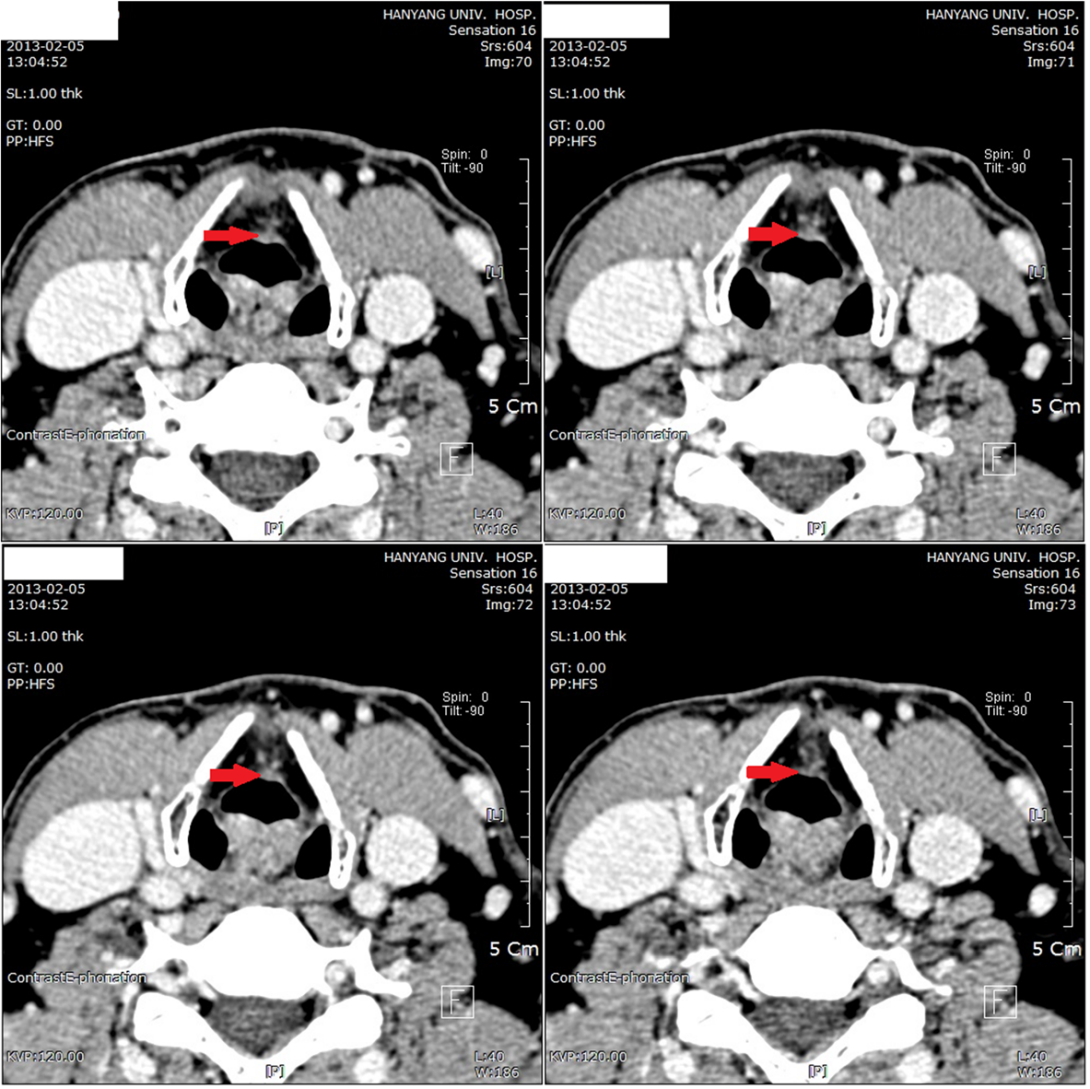
Neck 3-D CT/c CE (02/05/2013). It showed that the lesion had invaded the anterior commissure of the larynx but was limited to the right vocal cord with no invasion around the glottis

Present illness: The worker came to the Department of Otolaryngology Head and Neck Surgery, Hanyang University Hospital in February 2, 2013 complaining of hoarseness. Laryngoscopic examination revealed a lesion, an irregular surface, in his right vocal cord (Fig. 1). Neck CT carried out on February 5 showed that the lesion had invaded the anterior commissure of the larynx but was limited to the right vocal cord with no invasion around the glottis (Fig. 2).

Past history: Hypertension/Diabetes Mellitus/Tuberculosis/Hepatitis(−/−/−/−)

Social history: ex-smoker (20 pack-years), social drinker (1 bottle of so-ju per week)

Physical examination: Nonspecific findings

Pathologic findings: February 19, 2013, larynx, vocal cord, right, excision: Squamous cell carcinoma in situ

Radiologic findings:

February 4, 2013 Chest X-ray: no active lesion in the lung

February 5, 2013 Neck 3-Dimensional Computed Tomography with Contrast Enhancement:

Right vocal cord lesion – subtle enhancing lesion at the anterior vocal cord, with involvement of anterior commissure, and superficial lesion confined to vocal cord without evidence of paraglottic invasion.

Clinical course: Laryngoscopy was performed on February 19 for removal of both vocal leukoplakia, under suspension. Whole body PET-CT performed on March 6 showed no sign of metastasis, and type IV LASER cordectomy on both sides using a CO_2_ LASER with mitomycin application was performed on March 12. There was no sign of recurrence during follow-up ambulatory care.

Occupational history and working environment:

Occupational history: The worker started learning design and construction supervision at a construction firm in 1971 after graduating from high school at the age of 19 (Table 1). He joined a construction firm in March, 1977 and started to supervise construction regularly. He supervised both design and construction in subsequent workplaces and started to do only construction supervision from 1996. He worked as a supervisor for 38 years carrying out material inspection, site inspection and site management.

**Table 1:** Occupational history of worker (Korea Association of Construction Engineering & Management)

Working time	Working term	Workplace	Job description	Work type
1971. 2 ~ 1973. 3	2 years 2 months	Company C	Accounting demolition, inspecting materials, supervising the construction processes	Design & supervision
1976. 5 ~ 1977. 3	10 months	Company D		
1977. 3 ~ 1990. 1	12 years 11 months	Company U		
1990. 2 ~ 1995. 8	5 years 7 months	Company L		
1996. 2 ~ 1996. 5	4 months	Company Y		
1996. 6 ~ 2002. 7	6 years 2 months	Company U		Supervision only
2003. 1 ~ 2013. 2	10 year 1 month			
Total	38 years 1 month			

Workplace environment & work type: The worker inspected all materials stocked in the construction area. According to Material guide of the construction sites, The construction materials containing asbestos were astile (flooring material), asbestos-tex (ceiling material), bamlight (wall, ceiling material), insulator (asbestos fiber), outer wall material (ALC panel), etc. (Table 2). The inspected material was made up of asbestos-tex, astile, insulator, and bamlight.

**Table 2:** Asbestos content by construction material

Classification	Construction material	Asbestos content
Ceiling material	Asbestos-tex	White asbestos 4–6 %
Flooring material	Astile	White asbestos 5–15 %
Insulator	Asbestos gasket, tape	White asbestos 25–30 %
Wall, ceiling material	Bamlight	White asbestos 10–15 %
Outer wall material	ALC panel	White asbestos 10 %

After inspection, samples were taken and stored for subsequent matching of pattern and color. During inspection, the worker was exposed to material dust even if the material was new and dust always formed during construction in the field. When the supervisor finished inspecting some material, the material was passed on to the constructor for construction and the supervisor carried out a field inspection after construction was complete. In addition to this, site inspections were carried out frequently while working in the field. In the construction process, About 3 ~ 4 h of this worker’s working time for a day was spent for inspection.

**Fig. 3:**
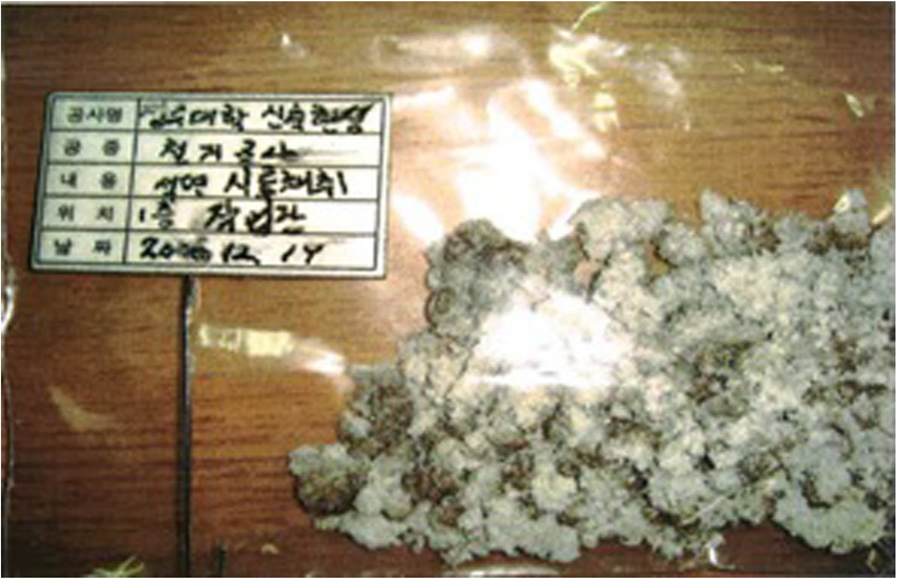
Asbestos sampling before demolition

**Fig. 4:**
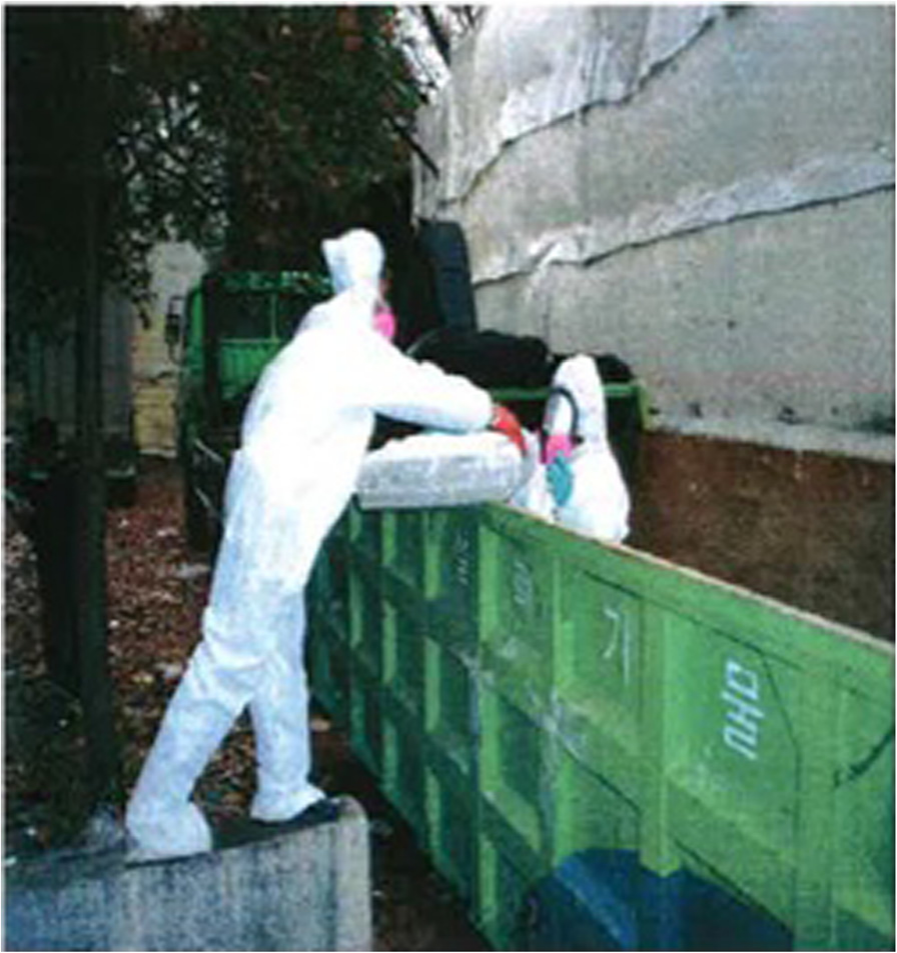
Disposal of asbestos waste after demolition

The worker in this case mostly supervised large scale construction sites where new buildings were erected after demolition of the previous buildings. He joined in the entire construction process: field inspection, demolition, fencing, and completing the new construction (Figs. 3 and 4). Most of the redevelopment projects supervised by the patient lasted more than a year, including demolition, and these accounted for 70 ~ 80 % of the total projects he had undertaken.

Asbestos has been regulated from 1990; it was first registered as a permitted material in the Korean Industrial Safety and Health Act but the use of asbestos-containing products (roof, ceiling, wall or flooring asbestos cement products) and asbestos friction products for automobiles was prohibited from 2006. Until then white asbestos in a low weight ratio was not prohibited but all asbestos-containing products including white asbestos started to be prohibited from 2009. Therefore we may assume that almost all buildings used asbestos-containing -materials in wall/ceiling, asbestos cloth, etc. during the working period of the patient.

Working environment measurement: No measurements were made due to the characteristics of the construction industry, and there was no related data. According to his words, Prior to 1996, it was either not equipped with protective equipment such as dust masks, or not actually wear.

## Discussion

There is not much data referring to the asbestos exposure situation in Korea and internationally. Also most of the data was concerns about asbestos exposure in asbestos mines, the textile industry, shipbuilding industry and automobile maintenance service rather than the construction industry. In the case of Dutch workers working in the asbestos cement industry, the groups working with the raw materials were exposed to an average of 7.5 f/cc of asbestos in the 1970s and the exposure level gradually decreased [13]. In a study of malignant mesothelioma, which is an indicator of asbestos exposure, the incidence rate was 5.8 times (95 % PI 5.3–6.5) higher in construction site workers [14]. In this case, because of absence of working environment measurement, we need to estimate asbestos exposure through exposure reconstruction using literature reviews. There are asbestos Exposure assessment studies according to industry. In the American workers, Considering the entire dataset that contained air sampling data from 1984 to 2011, not accounting for outliers, personal asbestos concentrations ranged from non-detectable (ND) to a maximum of 175 f/cc [15]. From 1984 to 1989, personal air samples of operative builder with detectable asbestos concentrations ranged from 0.001 to 16.1 f/cc. From 1990 to 1999, concentrations ranged from 0.003 to 13.2 f/cc, and from 0.0024 to 0.32 f/cc in the 2000s, respectively. Finally, from 2010 to 2011, personal air samples ranged in concentrations from 0.13 to 0.19 f/cc. In the Iran’s construction workers, during demolition of old house, Personal monitoring of asbestos fiber levels indicated a range from 0.01 to 0.15 PCM f/ml (0.02–0.42 SEM f/ml) [16]. In Korea, a study of Job exposure-matrix of asbestos was conducted by reviewing about literatures reported [17]. Construction industry was classified as third exposed group, in 1996 in 18 samples concentrations ranged from 0.01 to 0.32 f/cc (GM 0.05), in 2001 in the two samples concentrations were detected by 0.01 f/cc, in 2006 in the twelve samples concentrations ranged from 0.00 to 0.06 f/cc (GM 0.01) [17]. However, About the exposure study of building demolition workers which was expected to be exposed to a high concentration of asbestos, In 2002, concentrations ranged from 0.014 to 0.419 f/cc. In the case of the worker, because it is not enough to reconstruct job exposure matrix, we couldn’t estimate asbestos exposure dose. But, estimating asbestos exposure from historical data by year, from 1971 to 1995 there is no previous exposure data in the construction industry, so using previous exposure data in the shipbuilding and automobile maintenance services, we estimated minimum asbestos concentration ranged from 0.046 to 0.866 f/cc, from 1995 to 2000 concentrations ranged from 0.1 to 0.2 f/cc, after 2000, concentrations ranged from 0.01 to 0.05 f/cc. And during demolition that account one-third of the total construction process, it is estimated that asbestos concentration was twice to five times than that of construction process. Based on this, we estimated approximately that cumulative asbestos exposure of this worker was minimum 10 f/cc*year.

The worker in this case did not have pleural plaques or pleural thickening in chest X-rays. However, pleural plaques can be an indicator of asbestos exposure, they are not observed in all workers exposed to asbestos [18], and general radiographic examinations of the chest do not have high sensitivity or specificity [19]. This worker underwent endoscopy, biopsy and PET-CT so chest CT exam was not performed as it did not seem useful for the diagnosis.

Since smoking and drinking are the most important risk factors for laryngeal cancer, occupational risk factors have not been much studied in Korea. However, one factor identified in other countries is asbestos exposure, and others include exposure to diesel combustion material, polycyclic aromatic hydrocarbons (PAH), silica, welding fumes, nickel, sulfuric acid mist, rubber manufacturing, etc. and tree dust, formaldehyde, cement dust are known to make a small contribution [20–22].

Asbestos is a representative factor in relation to laryngeal cancer in an occupational and environmental medicine perspective. Various epidemiologic studies have examined about the correlation between laryngeal cancer and asbestos exposure. In a case–control study conducted in France on 1,833 patients with head and neck squamous cell carcinoma, asbestos exposure increased the risk of laryngeal cancer (OR 2.1, 95 % CI 1.6–2.8), and the risk increased with the length and severity of asbestos exposure [23]. Also in a study conducted on 307,799 Swedish industrial workers, 227 workers were diagnosed with laryngeal cancer, and asbestos exposure increased its incidence by RR 1.9, 95 % CI 1.2–3.1 [24].

However until recently there has been controversy over the relation between laryngeal cancer and asbestos exposure. In a Dutch study conducted on 58,279 patients, glottic cancer was correlated with organ exposure to asbestos, and supraglottic cancer was more strongly correlated [25]. However a meta-analysis of 69 studies referring to asbestos found no significant dose–response relationship between asbestos and laryngeal cancer [26].

Until 2009, the IARC accepted only limited evidence of asbestos being carcinogenic for laryngeal cancer. However in 2009, a report of a discussion about cancer and asbestos among 27 scientists from 8 countries held during an IARC was published in Lancet Oncology. In the report, it was suggested that there was adequate evidence that lung cancer, mesothelioma, ovarian cancer and laryngeal cancer could be caused by asbestos [12]. The IARC described the evidence in an IARC monograph (100C, 2012) citing cohort studies, case control studies, meta-analysis studies, etc.. In a meta-analysis of cohort studies by the Institutive of Medicine (IOM), the RR of the exposed group to the non-exposed group was 1.4 (95 % CI: 1.17–1.64), and the RR of the group exposed to high concentrations was 2.02 (95 % CI: 1.64–2.47) when applying the low standard, and 2.57 (95 % CI: 1.47–4.49) when applying the high standard (Some studies reported dose–response relationship on multiple gradient metrics. In computing the summary RR, “lower standard” calculation used the smallest “high vs. none” RR, and “high standard” calculation used largest “high vs. none” RR). In the meta-analysis of case control studies, the RR before adjusting for drinking and smoking was 1.43 (95 % CI: 1.15–1.78) and 1.18 (95 % CI: 1.01–1.37) after adjusting [27]. There is a need for additional quantitative exposure studies, such as studies of cumulative asbestos exposure, but it is now generally accepted that high concentration of asbestos or long term exposure to asbestos can cause laryngeal cancer in exposed occupational clusters.

The worker in this case worked as a supervisor for 38 years from the age of 19 in 1971 in material inspection, site inspection and site management. He did not handle asbestos directly but was constantly exposed to asbestos while inspecting asbestos-containing materials and working on site. In particular he participated in the entire process of demolition of previous buildings from site inspection to completion of demolition so he can be assumed to have been exposed to large amounts of asbestos. Therefore the laryngeal cancer which occurred in this worker is considered to be work-related.

This study has some limitations. First, in his occupational history before 1996, the worker’s activities in supervision and design were not defined. Therefore we could not calculate the precise exposure time. Second, we assumed that the risk factors for head and neck tumors and laryngeal cancer were similar, and this assumption needs more investigation. Third, level of the construction work environment was not measured, and past asbestos exposure was estimated from the statements of the worker. Fourth, there were no pleural plaques or pleural thickenings in the chest X-rays, so that exposure could not be proved by an objective standard. Finally, for this worker, we can’t rule out the laryngeal cancer due to other risk factors such as cement and silica in the workplace. Also, it could have been caused by the interaction between asbestos and other factors.

Despite these limitations, this study is the first case report suggesting a relation between exposure to asbestos and the development of laryngeal cancer in Korea. However, further epidemiological and experimental studies are needed in order to prove this connection definitively.

## Conclusion

The patient in this case worked with construction materials including asbestos and supervised construction for 38 years, and was thus exposed to asbestos at construction sites. Much of the asbestos was highly concentrated especially during demolition processes. We therefore consider the laryngeal cancer of this patient to be a work-related disease. The significance is that this study is the first case report suggesting a relation between exposure to asbestos and the development of laryngeal cancer in Korea.
